# Improved thermoelectric performance of Bi-deficient BiCuSeO material doped with Nb, Y, and P

**DOI:** 10.1016/j.isci.2021.103145

**Published:** 2021-09-16

**Authors:** Khabib Yusupov, Talgat Inerbaev, Mikael Råsander, Daria Pankratova, Isabella Concina, Andreas J. Larsson, Alberto Vomiero

**Affiliations:** 1 Institute of Physics, Chemistry and Biology (IFM), Linkoping University, 583 30, Linkoping, Sweden; 2 Sobolev Institute of Geology and Mineralogy SB RAS, Novosibirsk 630090, Russia; 3 L. N. Gumilyov Eurasian National University, Nur-Sultan 010008, Kazakhstan; 4 Experimental Physics, Division of Materials Science, Department of Engineering Sciences and Mathematics, Luleå University of Technology, 97 187 Luleå, Sweden; 5 Applied Physics, Division of Materials Science, Department of Engineering Sciences and Mathematics, Luleå University of Technology, 97 187 Luleå, Sweden; 6 Department of Molecular Sciences and Nanosystems, Ca’ Foscari University of Venice, Via Torino 155, 30172 Venezia Mestre, Italy

**Keywords:** Energy Resources, Thermal property, Energy materials

## Abstract

Thermoelectric materials convert waste heat into electric energy. Oxyselenide-based material, specifically, p-type BiCuSeO, is one of the most promising materials for these applications. There are numerous approaches to improve the heat-to-electricity conversion performance. Usually, these approaches are applied individually, starting from the pure intrinsic material. Higher performance could, however, be reached by combining a few strategies simultaneously. In the current work, yttrium, niobium, and phosphorous substitutions on the bismuth sites in already bismuth-deficient Bi_1-x_CuSeO systems were investigated via density functional theory. The bismuth-deficient system was used as the reference system for further introduction of substitutional defects. The substitution with phosphorous showed a decrease of up to 40 meV (11%) in the energy gap between conduction and valence bands at the highest substitution concentration. Doping with niobium led to the system changing from a p-type to an n-type conductor, which provides a possible route to obtain n-type BiCuSeO systems.

## Highlights

• Investigation of new semiconductor materials for thermoelectric application
• Study of electronic structure via density functional theory
• Prediction of new n-type semiconductor material
• Materials with enhanced heat-to-electricity conversion

## Graphical abstract

**Figure:**
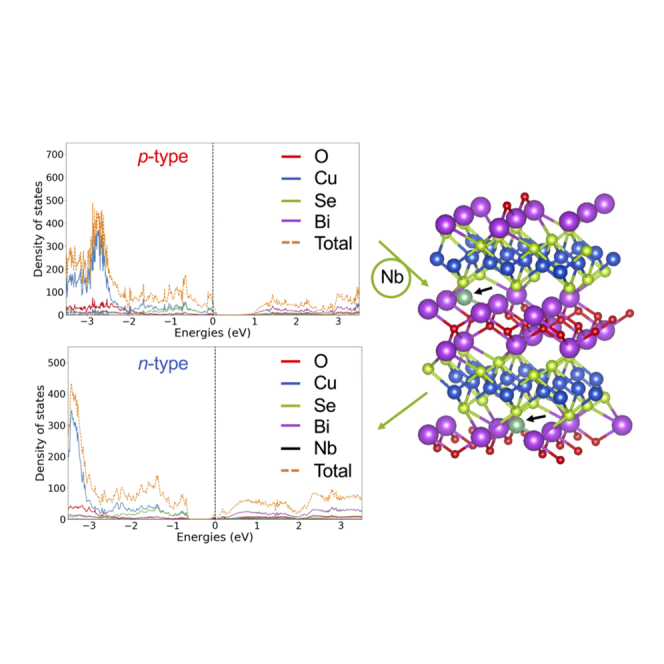

## Introduction

The modern society consumes large amounts of energy (Dong et al., 2018). Finding new and renewable energy sources is therefore a priority (Beretta et al., 2018; Blöchl, 1994). Waste heat is produced in all types of combustion and manufacturing. If one could make use of some of this heat, a significant step toward a more sustainable society would be achieved. Thermoelectric (TE) materials are used for their ability to convert heat energy into electricity. The process can be described simply as follows: the temperature difference created at the opposite ends of the material is leading to a potential difference which in turn creates a current flow within the system (Pourkiaei et al., 2019). The efficiency of TE material is determined by the dimensionless figure of merit $ZT=\;\left(S^{2}*\sigma /\varkappa \right)*T$, where S is the Seebeck coefficient,σ is the electrical conductivity, ϰ is the thermal conductivity, and T is the absolute temperature. For the efficiency to be high, one should increase the values in numerator, which is also called the power factor, while conserving or even decreasing the thermal conductivity in the denominator (Chen et al., 2020). The TE components can be useful in any field exhibiting a temperature difference, *e.g*., in smart electronics, wearable devices, medical equipment, cars, space satellites, and more (Jaziri et al., 2019).

TE materials are divided into three main groups according to their operating temperature: low-T (Yusupov and Vomiero, 2020), meaning operation between room temperature (RT) and 200°C, middle-T, operation between 200°C and 500°C, and high-T, operation from 500°C and above. The biggest part of the heat losses is in the region below 500°C which suggests that the region from RT to 500°C can be considered as the main target for the TE research field. In this specific region, polymer-based materials (Yusupov et al., 2018a, 2018b, 2020) and oxyselenides are considered among the most promising and newly developed materials due to their transport properties and air stability (Zhao et al., 2014). A recently developed material among the oxyselenides is BiCuSeO, which is a p-type semiconductor with an experimental indirect bandgap of about 0.8 eV (Hiramatsu et al., 2008). BiCuSeO exhibits high intrinsic values of the Seebeck coefficient (of 350 μV K^−1^) and low values for the thermal conductivity (ϰ) (0.6 W m^−1^ K^−1^ at RT), which is why the system attracts attention. It also exhibits a complex layered crystal structure with repeated 2D layers of Bi_2_O_2_ alternatively stacked with Cu_2_Se_2_ layers along the c-axis. It is suggested that the Bi_2_O_2_ insulating layer is responsible for the charge generation (GL), whereas the Cu_2_Se_2_ layer is responsible for charge flow and determines the conductivity (FL) (Ren et al., 2019b). The intrinsic values for the electrical conductivity are however rather low, which is the main disadvantage of the material and thus should be addressed to achieve high TE performance (Zhang et al., 2017).

To improve the properties of BiCuSeO, several approaches have been investigated. Li et al. (2015) have *e.g*. shown that a controlled introduction of vacancies leads to an enhancement of the figure of merit. The authors observed an increase in the electrical conductivity with a concomitant small decrease in the Seebeck coefficient, which resulted in a higher ZT (0.68) compared to the pure system. The study showed that the introduction of both Bi and Cu vacancies provides an improvement of the ZT. However, the authors also showed that the implementation of Cu vacancies is more difficult to control. And since Bi vacancies alone provide a high increase in the relevant properties, such can be considered to be a good strategy for future work.

Another approach to improve the electrical conductivity of BiCuSeO is doping with different elements. Lan et al. (2013) reported a divalent, lower oxidation state compared to the pure system when Bi was partially substituted with Pb (the oxidation state of Bi is +3 in the pristine BiO layer). Substitution of 6% of the Bi for Pb provided a significant increase of the electrical conductivity up to 135 S/cm, a decrease of the Seebeck coefficient down to 222 μV K^−1^, and a small insignificant increase of the thermal conductivity, which altogether led to a significant increase of ZT up to 1.14 at 550°C. The improvement is due to an increased charge carrier concentration and mobility when Pb is present within the system. Isovalent doping also leads to improved TE performance; however, the mechanism for this improvement differs. Feng et al. (2020) reported the influence of Sm doping on the TE properties of BiCuSeO. The incorporation of Sm *4f* orbitals strongly affected the band structure which led to a decreased bandgap, which in turn provided improved TE properties. The presence of Sm led to a higher electrical conductivity (∼79 S/cm) which resulted in enhanced ZT values of 0.74 at 600°C. As shown by Feng *et al.*, a decrease of the size of the bandgap is also considered to be a promising strategy for improving the TE properties of BiCuSeO and also that this can be achieved via isovalent substitutions.

Among the current goals of the TE field, there is one that deserves especial attention. The goal is to reach n-type semiconductor material with the equal TE performance to further build TE generators. Realizing n-type BiCuSeO is not an ordinary task. So far, it was achieved to some extent by the group of Lin et al. (Pan et al., 2018). In the work, it was demonstrated that substitution of Bi vacancies with Pb element alongside the substitution of Cu with Fe atoms can lead to obtaining of n-type material in the temperature region from room temperature to ∼450 K.

It has therefore been established that improvement of the TE properties of BiCuSeO can be achieved by introducing vacancies in the lattice and by substitutional doping on the Bi site. However, so far, no one has investigated the combined effect of vacancies and substitutions in BiCuSeO. Neither has anyone realized n-type conductive material with implementation via the combined effect.

In the current work, the combined effect of Bi vacancies and substitutions with various elements on the electronic properties of BiCuSeO was investigated, therefore building upon already established routes for improving the TE properties of the system. However, in the present study, we view the improvement of the TE properties from the presence of the Bi vacancies as our starting point and argue whether it is possible to further improve the TE properties with concomitant substitutional doping on the Bi sites. It was chosen to study the effects of substitutional doping of Bi with phosphorous (P), yttrium (Y), and niobium (Nb) as the substitutional elements. The reasons of why these elements were chosen are as follows: were not reported previously; abundant; easy to work with; and exhibit electronic configurations different from the Bi, hence could lead to the uncommon results. The effect of Bi vacancies and substitutions has been investigated using density functional calculations (DFT). It was found that each substitution leads to changes in the band structure and density of state (DOS) which provides an altered behavior in the system. The calculations reveal the possible improvement of the TE performance of BiCuSeO for all substitutions. Besides, a transition from a normal p-type semiconductor BiCuSeO system to an n-type semiconductor was found when doping with Nb.

### Computational details

**Figure 1:**
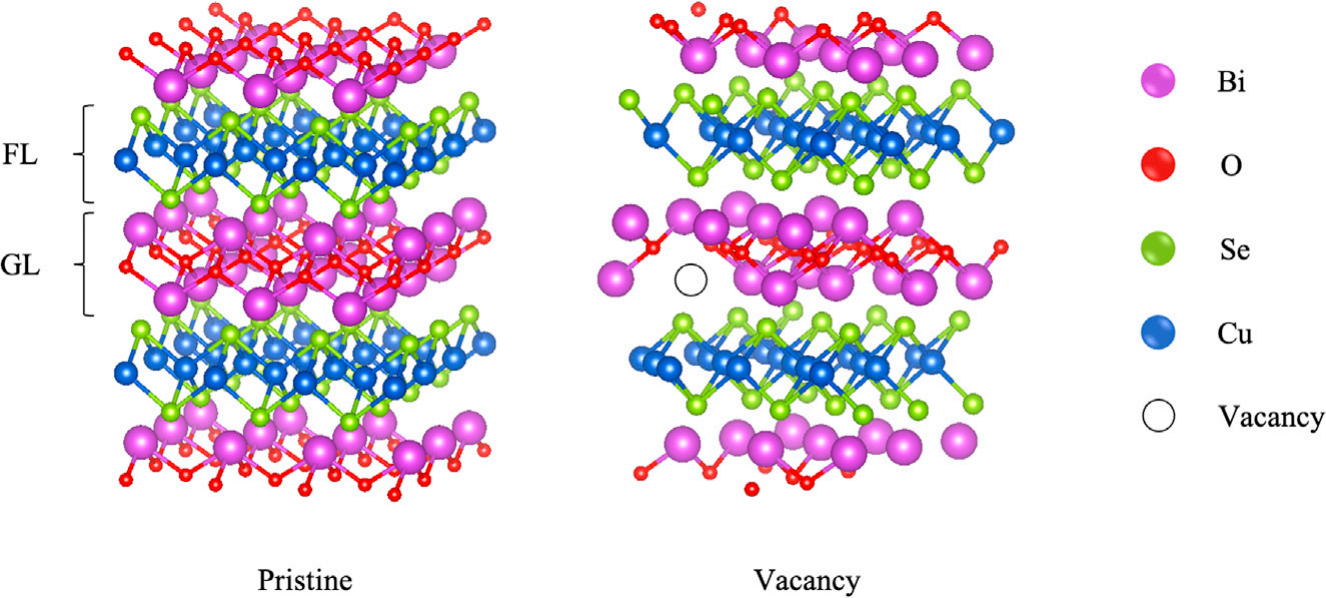
Structures of pristine and vacancy filled BiCuSeO Structures of pristine BiCuSeO crystal (left) and BiCuSeO system containing a single Bi vacancy (right): the view of the latter is rotated compared to the pristine case. The vacancy is represented by a hollow circle. GL and FL are charge generating and flow of charges layers, respectively.

The BiCuSeO tetragonal structure with space group *P4/nmm* was simulated using a 3 × 3×2 repetition of the primitive BiCuSeO unit cell, where the lattice constants of the primitive system were found to be ∼3.95, 3.95, and 9.04, respectively. The ideal supercell, *i.e*., without vacancies and substitutions, therefore contains 144 atomic positions. The introduction of a single Bi vacancy results in a system containing 143 atoms, refer Figure 1 (Structures of pristine BiCuSeO systems).

DFT calculations were performed using the projector-augmented wave method (Blöchl, 1994) as it is implemented in the Vienna ab initio simulation package (Kresse and Furthmüller, 1996). To account for the localization of specific atomic orbitals, especially regarding the localization of the Cu 3d states, the PBE+U method was used. The +U or Hubbard parameter was taken into consideration for each atomic species. Our tests showed that U only had a relevant effect on Cu. Therefore, an effective U = 4 eV was applied following previously reported studies (Zhang et al., 2011). All calculations were spin polarized, and spin-orbital coupling was considered. The plane-wave basis set energy cutoff was set to 520 eV. The electronic self-consistence loop was converged to 10^−6^ eV for all calculations. All structures were relaxed with no symmetry constraints using the conjugate gradient algorithm until all forces acting on the atoms were smaller than 10^−2^ eV/Å. For the supercell calculations, a 4 × 4 × 3 k-point mesh was used, whereas for the primitive cell, a 12 × 12×6 mesh was used. Analysis of charge localization and charge transfer was performed using Bader analysis (Yu and Trinkle, 2011).

## Results and discussion

The BiCuSeO system has been investigated previously by Fan et al. (2017), and they obtained a theoretical bandgap of 0.8 eV, which is close to the experimental reported value. They used the ordered primitive cell of the system with the utilization of a hybrid functional HSE06 calculation including spin-orbit coupling. This is an extremely time- and resource-consuming approach and is difficult to perform for larger supercells. In the present study where the influence of small concentrations of vacancies and dopants on the electronic properties of BiCuSeO has been investigated, DFT+U was used.

The crystal structure of the ideal BiCuSeO crystal is shown in Figure 1. The optimized structural parameters are in good agreement with previously published results (Barreteau et al., 2012) and are also in line with what is to be expected for these types of compounds (Råsander and Moram, 2015). As was mentioned in the introduction, the controlled incorporation of Bi vacancies has been found to improve the TE performance of the system. Bi vacancies were therefore introduced by removal of a single Bi atom in the supercell, as is also shown in Figure 1.

**Figure 2:**
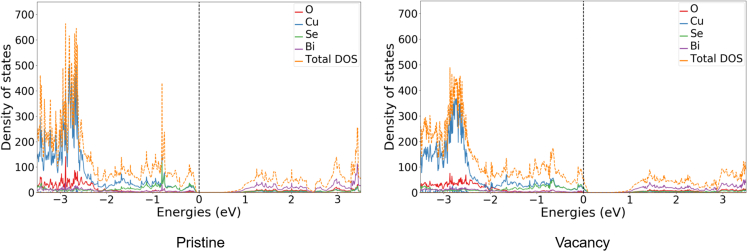
DOS of pristine and Vacancy filled BiCuSeO structures DOS of BiCuSeO. The pristine system (left) and the system with a Bi vacancy (right).

The DOSs of the ideal system and the system containing a single Bi vacancy are shown in Figure 2 (density of states for pure BiCuSeO systems). The presence of Bi vacancies shifts the Fermi level of the system into the valence band compared to the ideal system, which results in an increased DOS at the Fermi level. This is an indication of enhancing the system's p-type character. The change in the electronic structure is triggered by the removal of the Bi atom with the simultaneous relaxation of the nearby atoms. Overall, the DOS is not significantly changed in the presence of the Bi vacancy. The major change is the shift of the Fermi level and the subsequently increased number of states at the Fermi level.

**Figure 3:**
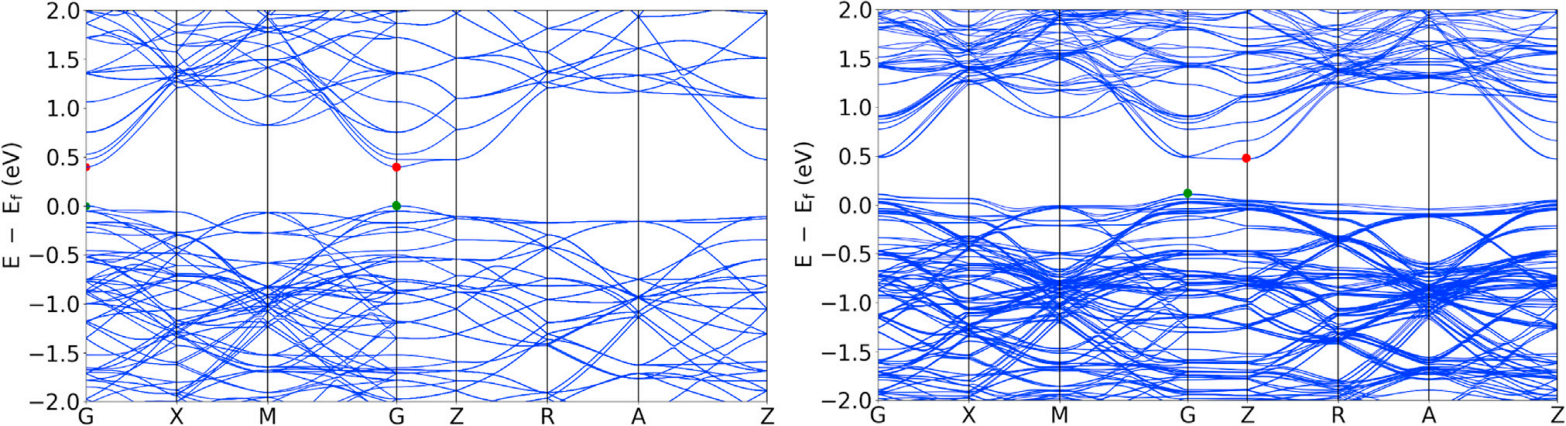
Band structures of pristine and vacancy filled BiCuSeO Band structure of pristine BiCuSeO (left) and the system containing Bi vacancies (right).

To obtain further insights into the electronic structure, the calculated band structures along the high symmetry directions in the Brillouin zone are shown in Figure 3 (band structures of pure BiCuSeO systems). In the current study, the change in the energy gap (*ΔE*) between the top of the valence band (VB), which is formed by the *d* states of Bi atoms, and the bottom of the conduction band (CB) (formed by the *p* states of Se atoms) will be discussed. The band structure of the ideal BiCuSeO crystal (Figure 3, left) exhibits a direct bandgap of 0.39 eV at the Gamma point. It should be noted that the bandgap is direct at the Gamma point for the supercell, while it would be direct and of the same size but at the Z point for the primitive cell. A similar direct bandgap though with a higher value (0.81 eV) was achieved by Fan et al. (2017) using the HSE06 hybrid functional, including spin-orbital coupling, which is essential for obtaining a direct bandgap for pristine BiCuSeO.

The introduction of vacancies changes the band structure so that the transition over the bandgap *ΔE* becomes indirect. This change is derived from a rather flat conduction band between Gamma and Z with a slightly lower value at the Z point. With Bi vacancies, the *ΔE* is also reduced in size from 0.39 eV to 0.36 eV; this alongside the increased number of bands around the Fermi level should give rise to increased electrical conductivity. For example, the *ΔE* of the Bi_2_Te_3_ is ∼0.5 eV, which results in a higher value of electrical conductivity (Mamur et al., 2018). Based on this comparison, one can assume that the change of electron structure should result in a similar outcome, *i.e*., increased values of the Seebeck coefficient. The introduction of Bi vacancies in BiCuSeO provides changes to the electronic structure that would favor increased TE efficiency.

**Figure 4:**
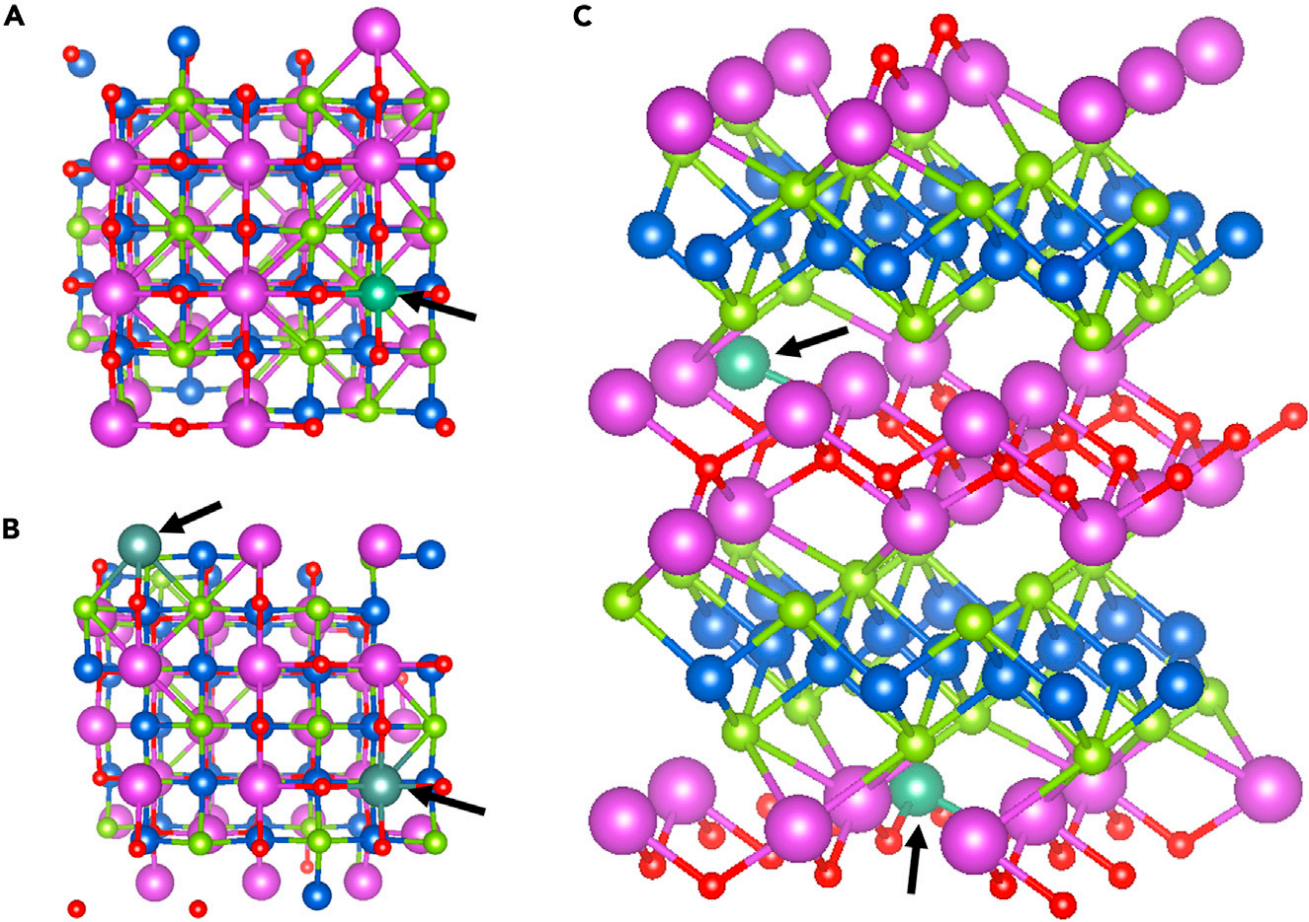
Structures of BiCuSeO with the doping positions Doping with Nb, P, and Y (A–C) (A) The placement of the doping element far from the vacancy, (B) placing two doping elements close to each other and far from the vacancy, and (C) placing two doping elements far from each other, one of which is near the vacancy. Black arrows are used to emphasize the doping positions. All models were used for each element substitution.

In the Bi vacancy system (Bi.Vc.), the stoichiometry is Bi_x_CuSeO with x = 0.97, which is used as the reference system for further investigations. Therefore, the substitutions on the Bi sites while at the same time having vacancies on the same lattice were investigated. The size of the supercell is such that the substitution of one or two Bi atoms will provide doping levels of 3% and 6%, respectively. Since any Bi atom is likely to be substituted, the calculations of structures with various positions for the doping elements were performed. Additionally, the positions of the dopants in relation to the position of the Bi vacancy were taken into account. In Figure 4, (structures of doped BiCuSeO systems) a representation of the structure from the *c* – axis point of view is showing the 3 at.% doping at a position far from the vacancy, Figure 4B shows 6% doping, and Figure 4C shows 6 at.% doping with the atoms far from each other and one of the doping atoms close to the vacancy.

The structures shown in Figure 4 have been investigated for each dopant. When comparing the total energies of the different configurations, we have found that for Nb and P at 6 at.% doping level, the most favorable configuration is the one shown in Figure 4C, i.e., the energy difference for the formation *b* and *c* is significant (for example, for Nb, the difference is ∼0.5 eV). In turn for Y doping at 6 at.%, the difference between *b* and *c* scenarios of doping is less than 0.01 eV, implying that the doping can be both near the vacancy and far from it. Considering this small difference, one could assume that the obtaining process of such material with Y doping can yield in the uneven distribution of the doping element within the system due to the small selectivity.

**Figure 5:**
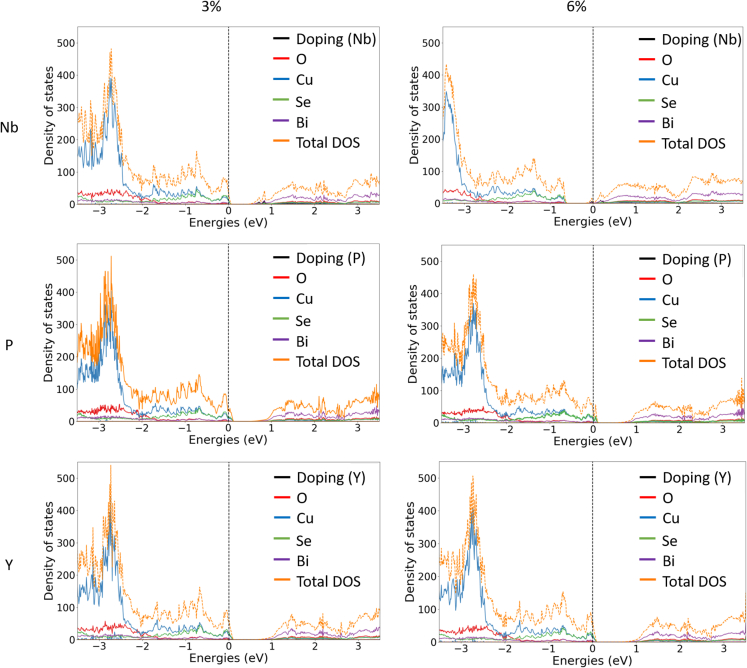
DOS of doped BiCuSeO DOS of Nb, P, and Y (row 1–3, respectively) doping concentrations of 3 and 6 at.% (left and right column) within the Bi vacancy Bi_x_CuSeO (x = 0.97) system. The x axis represents E–E_F_.

Figure 5 (density of states for doped BiCuSeO systems) shows the DOS for each dopant at both 3% and 6% doping. For 3 at.% Nb doping, the hole concentration in the valence band decreases and the conductivity gained by Bi vacancies is expected to drop. The overall doping effect of the system with Nb 3% leads to a restoration of the pristine BiCuSeO electronic structure, *i.e.,* DOS looks more like pristine BiCuSeO, which can be seen when comparing Figures 2 and 5. However, the introduction of Nb at 6 at.% leads to an n-type material since Nb is (Pei et al., 2013) an aliovalent substitution, and, in this case, each Nb injects two extra electrons into the system. The resulting n-type doping causes the Fermi level to be elevated into the conduction band. The Nb doping provides changes to the DOS of the CB, with clear defect level peaks close to the minima for both 3 and 6 at.%. For 6 at.%, the material has become an n-type semiconductor, but further electron promotion from VB to CB could also lead to a p-type character, like in the Bi.Vc. case (*cf*. Figure 2). Besides, an increase in the electron concentration results in higher values of electrical conductivity. Although the Seebeck coefficient and electrical conductivity are usually mutually excluding properties (Ge et al., 2016), the rise of both is possible due to doping if the system is not exhibiting a high concentration of charge carriers in the first place, which is the case for the Bi.Vc. system.

An increase of the doping concentration, which affects the nature of the semiconductor at higher concentrations, can lead to a transition between p-type and n-type nature (Zhang et al., 2015). That can be seen once the concentration of electrons is much higher than the concentration of holes within the system, leading to a change of contribution into a charge transport process. However, there are cases when even an insignificant concentration results in this type of transition (Bohra et al., 2018). In our case, this transition can be observed for 6 but not for 3 at.% Nb. At the 6 at.% concentration, the Bi.Vc. system exhibits n-type nature, *i.e*., the main charge carrier type is electrons. It should therefore be possible to make BiCuSeO n-type by the concomitant process of making the system Bi deficient while doping with Nb. In the past, research has mainly focused on the manufacture of p-type materials since they are more common. However, for successful utilization of the TE effect for a heat-to-electricity conversion, both types of semiconductors should be used (Champier, 2017). Nb doping is opening the possibility of using “one” material to create a full oxide TE generator with high TE performance and air stability.

Doping with the isovalent P did not result in a similarly dramatic change in the electronic structure. However, P has a smaller covalent radius, which results in a larger reconstruction of the lattice around the dopant. The DOS remains more or less the same as in the Bi.Vc. system. The doping with P leads to a small change in the Se and Cu DOS at the VB edge above the E_F_, especially at 6 at.%, which should lead to a small increase of holes and consequently higher electrical conductivity.

In the case of the isovalent Y doping, an unexpected behavior was detected. A very small shift of the Fermi level of 0.02 eV toward the VB edge (compared to the Bi.Vc. system) was observed, indicating slightly less p-type doping.

**Figure 6:**
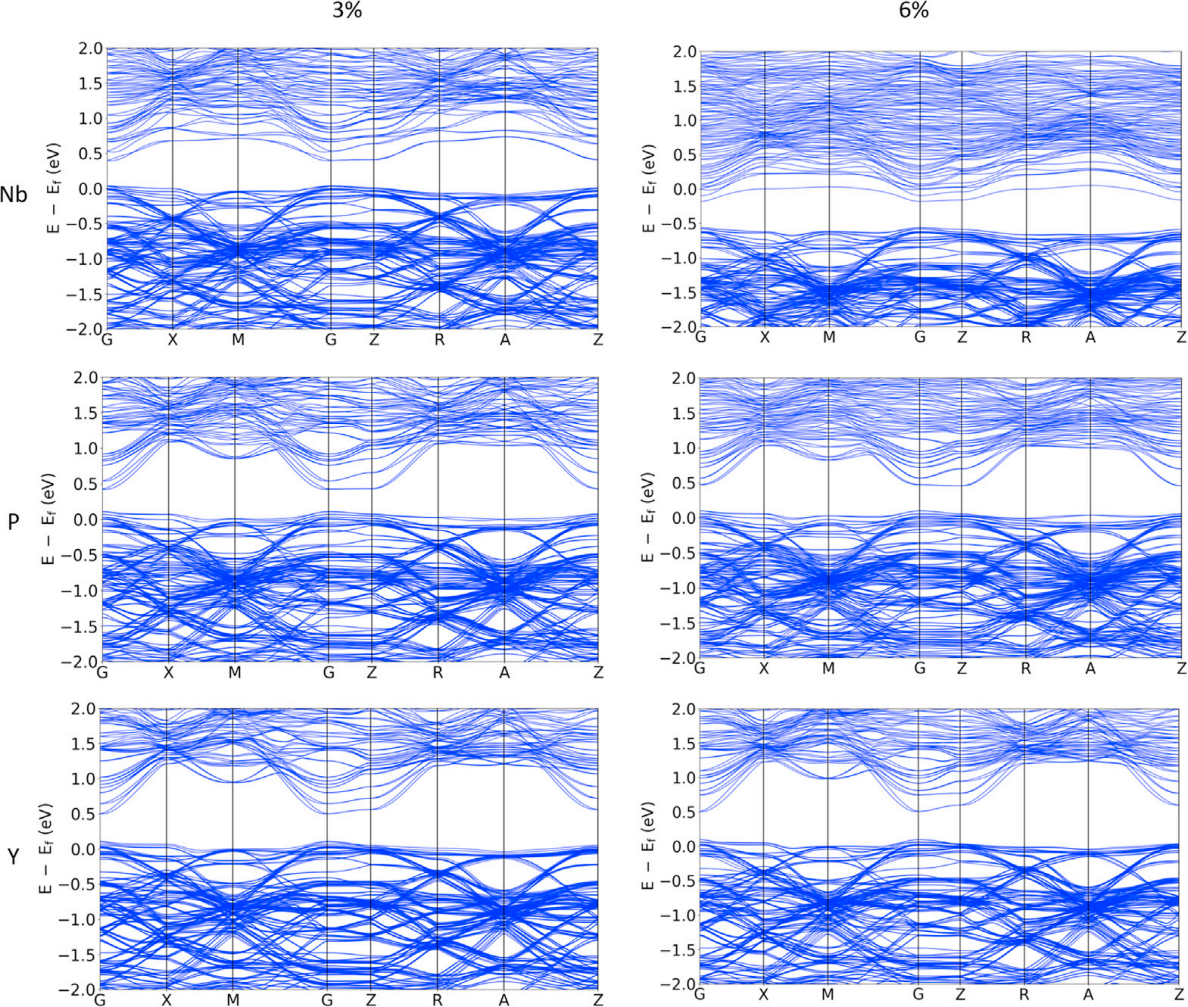
Band structures of doped BiCuSeO Band of Nb, P, and Y (row 1–3, respectively) doping concentrations of 3 and 6 at.% (left and right column) within the Bi vacancy Bi_x_CuSeO (x = 0.97) system.

The changes in the band structures with doping are presented in Figure 6 (band structures of doped BiCuSeO systems). The associated *ΔE* values for all dopant types and levels are listed in Table 1 (energy gap (ΔE) values for BiCuSeO-based systems). The doping with Nb leads to a decrease of the *ΔE* value (at doping of 3 at. %) compared to the vacancy system. The values of *ΔE* for 3 and 6 at.% of Nb doping are 0.35 eV and 0.38 eV, respectively. This is attributed to the defect levels introduced close to the CB edge, which forms shallow donor levels and shifts the Bi_2_O_2_ levels down in energy. This also makes the CB much flatter than in Bi.Vc. and the other doping systems. Further increase of defects leads to enlargement of the energy gap, which might be caused by the increased contribution of the doping elements. In this case, Nb exhibits higher energy states that form CB and hence lift the edge.

**Table 1:** Energy gap (ΔE) values for BiCuSeO-based systems

Systems	Pure	Bi.Vc.	Nb, 3%	Nb, 6%	P, 3%	P, 6%	Y, 3%	Y, 6%
*ΔE*, eV	0.39	0.36	0.35	0.38	0.32	0.35	0.39	0.41

The P doping affects the system in a different way, though the same trend of improved band density near the Fermi level and change of the *ΔE* are present. The *ΔE* values for 3 and 6 at.% are 0.32 and 0.35 eV, respectively. Since there are no dopant states close to either the VB or CB, the decrease of the *ΔE* values is explained by the differences in charge transfer and the smaller covalent radius of P atoms compared to the Bi (see below).

The Y doping influences the band structure differently. Firstly, instead of the reduction, an enlargement of the *ΔE* was observed. The increase is seen for both doping concentrations and the values of *ΔE* at 3 and 6 at. % are 0.39 and 0.41 eV, respectively. Also, in this case, there are no dopant states near the band edges, and the increase in *ΔE* is explained by secondary effects, such as differences in charge transfer and the larger covalent radius of Y compared to Bi, which is discussed below. Another distinctive effect of the Y doping is the change of the band structure pattern, *i.e.,* one can see that the band structure of the Y-doped system is similar to that of the pristine BiCuSeO (without the Bi vacancy) but with split bands, and a direct bandgap like in pristine BiCuSeO.

Doping of the Bi.Vc. system with Nb and P led to reduction of the *ΔE*, while Y doping led to an increase, which is not favorable for the targeted application. The Nb doping at 6 at. % concentration resulted in the change of nature from p-type to n-type conductivity.

The influence of doping on the charge per layer has also been computed to better understand possible changes to the transport properties. The sum of charges per layer from the atomic Bader charges is listed in Table 2 (charge per doping element in elementary charges (|e|).). The visual representation of the difference of charges per layer for each doping type is presented in Figure S2 for 3 at. % concentration. For simplicity, the Bi_2_O_2_ charge generating layers are labeled as GLs (1 and 2), and the charge flow layer Cu_2_Se_2_ is labeled as FLs (1 and 2). The GL layers are positively charged, whereas the FL layers are negatively charged.

**Table 2:** Charge per doping element in elementary charges (|e|).

Concentrations	Charge of the elements per doping scenario, eV				
	Bi	Nb	P	Y	Y (model b)
3%	1.55	2.34	1.92	2.10	–
6%	1.55	2.20	2.16	2.10	–
	1.58	2.38	1.89	2.11	–
6% (model b, Figure 4)	1.55	–	–	–	2.10
	1.54	–	–	–	2.10

Once the Bi vacancy is introduced into the layer GL2, the charge density decreases for this layer, and as a consequence, it can grant less charges to FL2 (and to a minor degree FL1). This asymmetry in charge donation can be associated with the position of the atom/vacancy. The layered structure of the BiCuSeO system is even more complex due to the presence of two atomic sublayers in each BiO and CuSe layers. Considering this, one can conclude that the closer the removed atom is to a specific layer, the more this precise layer should be affected by such “neighbor”. This is likely the cause of the shift in the Fermi level from the VB edge into the VB, which is located on the Cu_2_Se_2_, as seen in Figure 2. This verifies that Bi vacancies causes hole formation in the material. The vacancy introduction leads to that there is one less Bi atom that can contribute into the electronic cloud. As the result of it, there is decreased electron donation from the Bi atom to FL layers. The donation is thus asymmetric from these neighboring layers, and the effect on the FL1 layer also influences the GL1 layer; the exchange between these layers decreases to 0.09 less |e|. Further, the influence of doping with Nb, P, and Y on the charge of the layers is compared with the Bi.Vc. system below.

Doping with Nb leads to an increase of the asymmetry (system becomes less symmetric charge wise) of the charges between the layers caused by the vacancy introduction. The increased asymmetry and the charge for GL1 layer are explained by a higher atomic charge of Nb atom (2.34 eV) (Table 2) compared to the Bi atom (1.55 eV) that was substituted. The different degrees of change in nearest FL1 and FL2 layers can be explained with the same logic as the influence of vacancy above. The changes are dictated by the placement of the substitution atom, i.e., Nb atom is placed closer to the FL2 layers, hence higher electron donation is observed for the layer compared to the FL1. In addition, Nb has a smaller covalent radius than Bi, and the doping leads to distortion of the crystal. The shorter Nb-O bonds (see Figure S1), which also affects the shift of the levels in the CB. The distances between the adjacent atoms compared to the Bi.Vc. system are shown in Figure S1 for the 3 at.% doping concentration. A graphical representation of the adjacent bond lengths is shown in Figure S1. The effect of asymmetry is partially reversed for the increased Nb concentration of 6 at.%, i.e., placement of the second Nb atom in GL2, where the vacancy is located. The increased concentration of the doping element leads to a lower charge at first Nb atom (to 2.2 eV), whereas the charge at the second Nb atom exhibits a higher value (2.38 eV). The described changes in the charges of doping atoms are the reason for a dramatic enhancement of charge for GL2 layer.

The doping with P decreases the charge of GL1 layer, which is due to the low atomic charge of P (1.92 eV) and increases the asymmetry of the charge distribution. Though the charge of P is higher than that of Bi, but due to its rearrangements within the GL layer, since the FL2 layer receives less charges from the GL1 layer; the FL1 layer, on the other hand, receives more charges compared to the system with vacancy, which is probably due to the compensation of the charge. Further increase of the concentration leads to the electron’s attraction to the GL layers and to even higher restrictions of the electron donation to the FL layers. Hence, the values are decreasing for the nearest layers.

The doping with Y, as was mentioned before, is a special case since there is almost no difference in total energy of the system between formations *b* and *c* of Figure 4. At 3 at. % doping concentration, the mainly affected layers are GL1 and FL2, which is due to the high value of atomic charge of Y 2.10 eV. The FL2 layer is again receiving more charges than that of FL1 due to the position of the atom. The larger radius of Y caused crystal distortions, which results in opposite behavior of Bi-O bonds around the dopant compared to the Nb and P doping. Further increase of doping concentration is considered for two cases:

Single Y atom in each GL layer (Table 3 [Charge per layer for each system in elementary charges (|e|).]). In this scenario, the extra dopant mainly affects the GL2 layer, whereas GL1 remains almost the same as for 3 at. % concentration. The increase of the charge observed for FL1 layer is due to the donation of electrons from GL2 layer, i.e., Y atom is placed in the upped sublayer and hence closer to FL1 layer. It should be noted that the influence of one Y in each layer diminishes the charge asymmetry effect seen with only doping in GL1 for the lower doping concentration. Hence, increase of the doping concentration reduces the changes seen for the charges.

**Table 3:** Charge per layer for each system in elementary charges (|e|)

Charge transfer data, V									
Name of layer	Pure	Bi.Vc.	Nb doped		P doped		Y doped		
			3%	6%	3%	6%	3%	6%	6%
GL1	8.473	8.382	8.839	8. 672	8.134	8.104	8.663	8.666	8.945
FL1	−8.473	−8.136	−8.257	−8.381	−8.252	−7.925	−8.118	−8.382	−8.080
GL2	8.473	7.385	7.417	7.933	7.382	7.151	7.393	7.613	7.396
FL2	−8.473	−7.632	−7.999	−8.223	−7.264	−7.330	−7.939	−7.919	−8.261

Black circles represent the position of substituting atoms in various layers, whereas the empty circle represents the position of the vacancy.

Both Y atoms are in the GL1 layer. In this case, the increased doping affects the charge in the GL1, FL1, and FL2 layers in a more pronounced way, *i.e*., the difference in values between 3 and 6 at.% concentrations is higher than that in the first case. The influence in GL2 is similar to the value at 3 at% concentration. The increase and decrease of the values for FL2 and FL1 are due to the position of Y atoms, which are closer to the FL2 layer leading to the increased donation to this layer and lower share of charges with FL1 layer, respectively. The GL1 change is dictated by the presence of both Y atoms and hence increased density of atoms with higher charge.

The doping affects the crystal and electronic structure differently. The doping with Nb gives the largest impact on the electronic structure due to the extra valence electrons, changes in charge transfer, and smaller size, and increased doping even changes the type of conductivity along with balancing the charge imbalance in layers caused by introduction of Bi vacancies. The changes in crystal structure are less significant compared to the doping with two other types of doping, due to similar values of covalent radius of Bi and Nb. Doping with P gives the largest decreases in the band gap, increases the charge asymmetry between the layers, and gives the largest distortion of the crystal structure. However, it affects the electronic band structure in a lesser way. The incorporation of Y is quite different, with increased band gap, reduction of the charge asymmetry in the layers, and affects the structure in an opposite way due to the bigger covalent radius than Bi. The distortions of the structure caused by doping will further affect the phonons.

The impact of doping with Nb, P, and Y on the TE properties for BiCuSeO-based materials is discussed below. As was mentioned above, the introduction of Bi vacancies leads to an improvement of both the Seebeck coefficient and the electrical conductivity (Li et al., 2015). This alteration of the crystal structure, position of the Fermi level, and layer charge asymmetry leads to a reduction of the *ΔE* alongside a split of the bands due to the asymmetry of the crystal. Considering these changes and the influences described in the experimental study, we deduce that the doping causes certain improvements of TE performance.

### Influence on electrical conductivity

The Nb incorporation leads to a decrease of *ΔE*, further increases the structural asymmetry, and leads to improved donation of electrons to the CuSe layers, which together should increase the electrical conductivity. However, a further increase of doping enlarges the *ΔE* and changes the conductivity from p- to n-type. This points toward a new n-type BiCuSeO-based semiconductor and the possibility of building a full-oxide oxyselenide TE generator.

The doping with P results in enhanced asymmetry both with regard to structure and layer charge. It decreases *ΔE* that can lead to increased values of electrical conductivity, as also the charge asymmetry could improve charge transport. Overall, the strain within the structures due to Bi vacancies and substitutional doping should lead to improving the conducting performance of these p-type oxyselenide semiconductors since the holes generated by Bi vacancies pertain with the P-doping.

Also for Y-doping, the holes generated by introduction of Bi vacancies are retained, which should be the most important factor for improvement of the electrical conductivity. However, the doping with Y affects the properties differently since the substitution led to an increased value of *ΔE* and smaller Bi-O bonds around the dopant. The increased *ΔE* should generally lead to the decrease of the TE performance; however, the enhanced asymmetry and affinity for same layer doping could be beneficial. The GL layers are responsible for the transport of holes, and the case where the FL layers remain intact also when doped is certainly desired. The latter is reasoned by the decrease of the charge asymmetry and the similar appearance of the band structures between Y-doped and pristine BiCuSeO.

### Influence on thermal conductivity

In general, one expects an increase of the transport properties for the systems considered in this study; however, it might as well improve the thermal conductivity. This property consists of two main parameters: electronic (ETC) and lattice (LTC) transport of heat (Beretta et al., 2018). Though the increase of the electronic contribution can raise the overall thermal conductivity, the lattice part can diminish this influence and partially cancel it out. It should be noted that the main contribution to the overall thermal conductivity comes from LTC, whereas the ETC slightly affects it. Doping with the atoms of different sizes can result in a decreased LTC contribution (Ren et al., 2019a). According to Kumar and Schwingenschlögl (2016), the direction of thermal conductivity in BiCuSeO crystal structure is perpendicular to the layers. They estimated that atomic substitutions lead to phonon scattering, due to changes in the vibrational modes, hence decreasing the phonon contribution to thermal conductivity, but this needs to be verified. Based on our simulations and their estimates, one can expect a decrease of the overall thermal conductivity due to the diminished contribution of LTC, though with partial cancellation due to the expected increased contribution from ETC. The latter is specifically true for Nb doping, where the change from p-type to n-type conductivity is observed.

### Influence on Seebeck coefficient

The Seebeck coefficient highly depends on the electronic and thermal conductivities of the material. Depending on how big the change one acquires for those two properties, one can predict certain behavior of the Seebeck coefficient. In general, the coefficient becomes smaller with higher ϰ and tends to decrease with smaller values of thermal conductivity. However, if the increase of σ is dramatic, one can observe a decrease in the S values (Li et al., 2015). It can be concluded that with decreased values of ϰ (predicted above), the values of Seebeck coefficient should increase leading to a higher TE performance of the both p- (P, Y) and n-(Nb) type doping of the semiconductor.

In summary, we predict that doping with Nb, P, and Y alongside introduction of Bi vacancies should raise the overall ZT through increasing the numerator parameters and keeping constant or negligibly improving the denominator k parameter.

### Conclusions

In the current work, the influence of niobium, yttrium, and phosphorous doping alongside Bi vacancy presence on the crystal and electronic structure and TE properties was investigated. It was estimated that doping with any of the elements leads to n-type doping tendency compared to the Bi vacancy filled system. The doping with Nb and P at 3 at.% concentration leads to the decrease of the *ΔE* values due to the covalent radius of the doped elements, with further growth of the *ΔE* at increased concentrations, which is due to the changed distance between the layers. For Nb doping, at 6 at.%, the transition of nature from p-type to n-type semiconductors is observed. This transition is due to the oxidation state of the niobium, *i.e*., the doping is aliovalent with extra electrons. This doping creates extra states at the bottom of CB. The incorporation of yttrium leads to the widening of the *ΔE* with a distinctive feature that the substitution of Bi atoms can be in any of the GL layers. The DOS of the investigated systems exhibits densification near the Fermi level and distortions of the crystal structures caused by the covalent radius of the doping elements which increases asymmetry of the electronic structure of oxyselenide. Conducted research provides an explicit and thorough investigation of the simultaneous application of two strategies for improvement of TE performance for the p-type system (doped with Y and P) and achievement of n-type BiCuSeO-based semiconductor by Nb doping at a low concentration.

### Limitations of study

Provided theoretical results are limited by the used pseudopotentials. Displayed results regarding the Fermi level values and band gaps should be recalculated using hybrid functionals if one intends to acquire actual values.

## STAR★ Methods

### Key resources table

**Table:** 

REAGENT or RESOURCE	SOURCE	IDENTIFIER
**Software and algorithms**		

VASP package 5.4		https://www.vasp.at
Python version 3.9	Python Software Foundation	https://www.python.org

### Resource availability

#### Lead contact

Further information and requests for resources and reagents should be directed to and will be fulfilled by the lead contact, Khabib Yusupov (khabib.yusupov@liu.se).

#### Materials availability

All data reported in this paper will be shared by the lead contact upon request.

### Method details

#### Computational details

The BiCuSeO tetragonal structure with space group *P4/nmm* was simulated using a 3×3×2 repetition of the primitive BiCuSeO unit cell, where the lattice constants of the primitive system were found to be ∼3.95, 3.95, and 9.04, respectively. The ideal supercell, *i.e*., without vacancies and substitutions therefore contains 144 atomic positions. The introduction of a single Bi vacancy result in a system containing 143 atoms, see Figure 1 [Structures of pristine BiCuSeO systems].

DFT calculations were performed using the projector-augmented wave method (Blöchl, 1994) as it is implemented in the Vienna ab initio simulation package (Kresse and Furthmüller, 1996) (VASP). To account for the localization of specific atomic orbitals, especially regarding the localization of the Cu 3d states, the PBE+U method was used. The +U, or Hubbard parameter, was taken into consideration for each atomic species. Our tests showed that U only had a relevant effect on Cu. Therefore, an effective U = 4 eV was applied following previously reported studies (Zhang et al., 2011). All calculations were spin-polarized and spin-orbital coupling was considered. The plane-wave basis set energy cutoff was set to 520 eV. The electronic self-consistence loop was converged to 10^-6^ eV for all calculations. All structures were relaxed with no symmetry constraints using the conjugate-gradient algorithm until all forces acting on the atoms were smaller than 10^-2^ eV/Å. For the supercell calculations, a 4×4×3 k-point mesh was used, whereas for the primitive cell a 12×12×6 mesh was used. Analysis of charge localization and charge transfer was performed using Bader analysis (Yu and Trinkle, 2011).

## Data Availability

Used in this research The Vienna Ab initio Simulation Package is available through the license. This paper does not report original code.
