# Supplementary material for: Improved thermoelectric performance of Bi-deficient BiCuSeO material doped with Nb, Y, and P
Source: iScience. 2021 Sep 16;24(10):103145. doi: 10.1016/j.isci.2021.103145 (PMC8531848; doi:10.1016/j.isci.2021.103145)
Supplement: Document S1. Figures S1 and S2 [file mmc1.pdf]

iScience, Volume *24*

Supplemental information

Improved thermoelectric performance of Bi-deficient BiCuSeO material doped with Nb, Y, and P

Khabib Yusupov, Talgat Inerbaev, Mikael Råsander, Daria Pankratova, Isabella Concina, Andreas J. Larsson, and Alberto Vomiero

Supplementary information

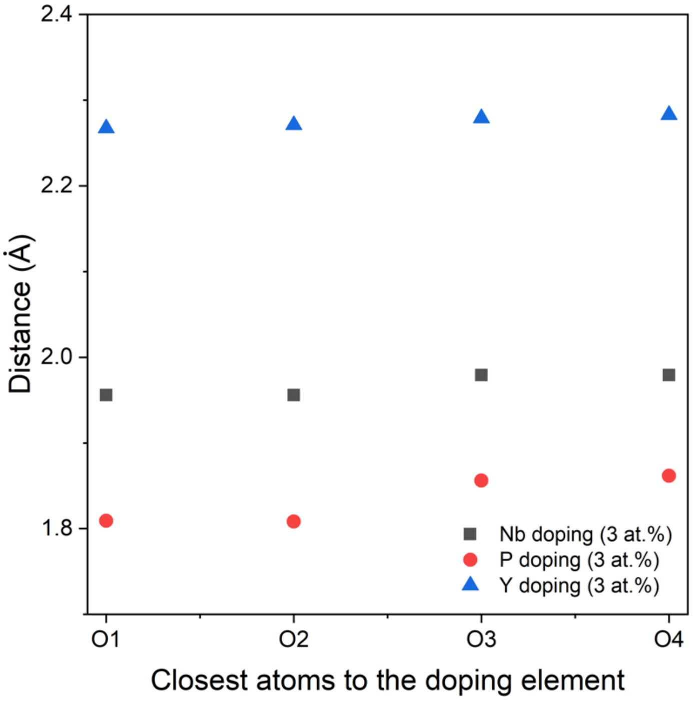

Figure. S. 1. Distance between doping elements (Nb, P, Y) and adjacent oxygen atoms at doping concentration 3 at.%. The doping element is placed at the 0 position and used as the reference atom. Related to table 2.

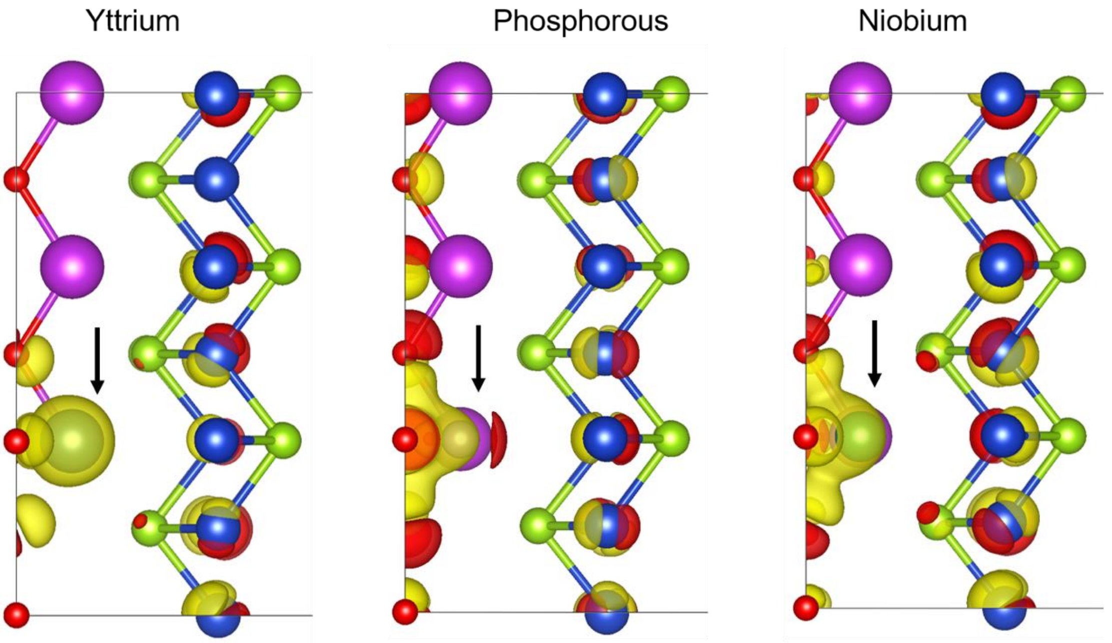

Figure. S. 2. Charge density difference for Nb, P, and Y doping at 3 at.% with comparison to Bi.Vc. system. The position of the doping element is emphasized by the black arrow. The main difference is represented by the HL layer (Bi_2O_2) and the closest CL layer nearby (Cu_2Se_2). Yellow and red colours of the densities represent negative and positive charges, respectively. Related to table 3.
